# Soil N Fluxes in Three Contrasting Dry Tropical Forests

**DOI:** 10.1100/tsw.2001.383

**Published:** 2001-11-20

**Authors:** Naoko Tokuchi, Asami Nakanishi, Chongrak Wachirinrat, Hiroshi Takeda

**Affiliations:** Laboratory of Silvicullture, Graduate School of Agriculture, Kyoto University, Japan

**Keywords:** N flux, dry tropical, mineralization, N uptake, N leaching

## Abstract

A comparative study of N fluxes in soil among a dry dipterocarp forest (DDF), a dry evergreen forest (DEF), and a hill evergreen forest (HEF) in Thailand was done. N fluxes in soil were estimated using an ion exchange resin core method and a buried bag method. Soil C and N pools were 38 C Mg/ha/30 cm and 2.5 N Mg/ha/30 cm in DDF, 82 C Mg/ha/30 cm and 6.2 N Mg/ha/30 cm in DEF, and 167 C Mg/ha/30 cm and 9.3 N Mg/ha/30 cm in HEF. Low C concentration in the DDF and DEF sites was compensated by high fine soil content. In the highly weathered tropical soil, fine soil content seemed to be important for C accumulation. Temporal and vertical fluctuations of N fluxes were different among the sites. The highest N flux was exhibited at the onset of the wet season in DDF, whereas inorganic N production and estimated uptake of N were relatively stable during the wet season in DEF and HEF. It is suggested that N cycling in soil becomes stable in dry tropical forests to intermediate in temperate forests. N deposition may result in large changes of N cycling in the DDF and DEF due to low accumulations of C and N.

### INTRODUCTION

Tropical dry forests represent more than 40% of tropical forest areas, but little study has been done in these dry forests with respect to atmospheric deposition of N[1,2,3]. Furthermore, very few of the studies about N budget in forest ecosystems relate to temporal and spatial patterns of N cycling in the tropical regions[4,5]. Knowledge of N cycling is essential for understanding the N status of ecosystems and the response of such systems to global changes in N deposition. The objective of this work was to detect the temporal and vertical patterns of N fluxes in a dry tropical region.

### METHODS

#### Study Sites

This study was conducted at the Sakaerat Environmental Research Station (SERS), in the southeast fringe of the Korat Plateau (14° 31′N, 101°55′E) in northeastern Thailand. The Sakaerat Environmental Station encompasses 81 km$^2$ of a reserved forest. Forests in Thailand are generally classified into six types as follows: hill evergreen forest (HEF), dry evergreen forest (DEF), dry dipterocarp forest (DDF), mixed deciduous forest (MDF), tropical evergreen forest (TEF), and mangrove forest[6]. DEF and DDF have characteristics that are typical of dry tropical forests.

The dry season in this area lasts from November to May, whereas the wet season follows from May to November. The total precipitation was 1296 mm in DEF and 1616 mm in DDF in 1999. The annual mean temperature was 26°C. The DDF is located at an elevation of about 300 m and the DEF is located at an elevation of 520 m, but the sites were nearly flat topographically. In SERS, the soils are underlain by sandstone[7]. Dominant tree species were *Hopea ferrea* and *Shorea henryana* in DEF[8], and *S. obtuse* and *Xylia xylocarpa* in DDF[9].

HEF was studied at Kog Ma forest watershed in Chiang Mai (18° 47' N, 98° 59' E). Kog Ma forest watershed is in a mountainous area with an elevation of 1200 m. Parent material is gneiss and schist. The dominant tree species in the watershed are *S. henryana* and *H. ferrea.* The annual mean precipitation in the area was 2055 mm and annual mean temperature was 26°C in 1999. A study transect was set up along a 120-m slope in HEF. In order to take into account topographical effects in our study, we made ten subplots at 10-m intervals along the slope transect. The toe slope experienced aquatic conditions during the wet season. Thus, we used nine of the subplots that were not saturated.

### Soil Sampling and Sample Preparation

The study plots were set up in 1998. During sampling, the litter layer was removed and the soils were sampled and replicated five to nine times at each site. Soil samples were obtained by using 100-cm$^3$ (5-cm diameter) core samplers to collect samples at the following depths: 0 to 5 cm, 5 to 10 cm, and 10 to 30 cm. Soil sampling was done from September 1998 to November 1999 at the onset of the wet season (May 1999), the middle of wet season (September 1999), and the late wet season (November 1998 and November 1999). Soil samples were transported to the laboratory with ice packs and were processed within 24 h of collection. The soil samples were sieved through a 2-mm sieve to remove roots and to homogenize the samples. The samples were then refrigerated for further analyses. Subsamples from each subplot were dried, sieved, and used for calculating the fine soil content. The fine soil content was used to express the fluxes and potential values on an area basis. Moisture content was determined for each sample by oven drying for 48 h at a temperature of 105°C.

### Total C and N

Total C and N of the oven-dried ground samples were determined by using a Nitrogen-Carbon analyzer; 5 g of soil were extracted by shaking with 50 ml of 2 *M* KCl for 1 h. The soil extracts were filtered and used to assess the pool size of inorganic N as $NH_4^+$ and $NO_3^-$. Ammonium was measured by the indophenol blue method[10]. Nitrate and nitrite were determined after zinc reduction instead of Cd reduction[10]. Concentrations were calculated to an area basis by multiplying the fine soil contents.

### Ion Exchange Resin Core

Ion exchange resin was used to adsorb $NH_4^+$ and $NO_3^-$ from soil solution[11,12,13]. For estimating N flux on an area basis, the 5-cm diameter and 1-cm length core were filled with ion exchange resin. The resin core was prepared by adding 15 g of cation resin (IR-120, Organo Chemical Company) and 15 g of anion resin (IRA-400, Organo Chemical Company). The upper side of the core was covered by 1-mm mesh nylon, whereas the bottom side of the core was covered by 0.02-mm nylon mesh for preventing resin leaching. The amount of ion resin in the core was thought to be enough to adsorb the N flux during the period[13]. The ion exchange resin cores (IER-core) were placed at 5-, 10-, and 30-cm depths at each subplot in May of 1999. The IER-core was exchanged in May (onset of wet season), September (middle of wet season), and November (onset of dry season). The IER-core was collected four times: September to November 1998, November 1998 to May 1999, May to September 1999, and September to November 1999.

### *In Situ* N Mineralization — Buried Bag Method

The buried bag method was used for estimating *in situ* N mineralization[14]. Fresh soil cores were sampled with 100-cm$^3$ soil cores from the subplots at each site. Soil samples collected at 0- to 5-, 5- to 10-, 10- to 20-, and 20- to 30-cm depths were covered with polyethylene bags with minimal disturbance. The buried bags were installed at the same depths of sampling and incubated *in situ* from November 1998 to November 1999. The bags were replaced as follows: between November 1998 to May 1999 for the dry season; between May to September 1999 for the early wet season; and September to November 1999 for the late wet season. Most of the long incubation period (about 6 months) was during the dry season in this area, where the antecedent soil moisture does not vary widely. After the *in situ* incubations, the buried bags were sieved through a 2-mm sieve, and extracted with 2 *M* KCl. The extracts were filtered and frozen until analysis. Ammonium was measured by the indophenol blue method[10], whereas nitrate and nitrite were determined after Zn reduction instead of Cd reduction[10]. Net *in situ* mineralization was calculated as the difference between pre- and postincubation concentrations of $NH_4^+$ plus $NO_3^-$. Net *in situ* nitrification was calculated as the difference between pre- and postincubation concentrations of $NO_3^-$.

Inorganic N production rates were calculated from the *in-situ* buried bag method, and inorganic N leachate from the soil horizon was determined from the ion exchange resin core method. The N uptake was estimated as follows:

$$\begin{aligned}\text{Estimated } N_{\text{uptake}} = {} & N_{\text{Input (production + rainfall or leachate from upper horizon)}} \\ & - N_{\text{Pool size change (inorganic N pool at the previous sampling – inorganic N pool at the sampling)}} \\ & - N_{\text{Output (leachate to the lower soil horizon)}}. \qquad (1)\end{aligned}$$

The method was modified according to Nadelhoffer et al.[5]. Statistical analysis was done using the one-way ANOVA.

## RESULTS

### C and N Accumulation

The C and N concentrations were highest in the surface soil horizon and decreased with soil depth in all sites (Fig.1). The de-

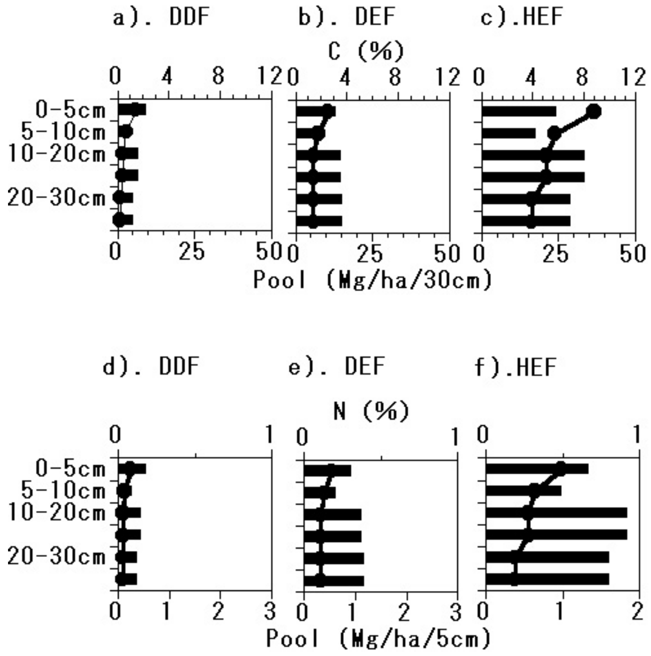

**FIGURE 1.** C and N accumulations in soil (0- to 30-cm depth). Bars are C and N accumulation amounts and lines are concentrations.

crease of concentrations was more drastic in DDF than HEF. Soil C and N pools increased from DDF, DEF, to HEF, 38.3 and 2.5 Mg/ha/30 cm in DDF, 82.2 and 6.2 Mg/ha/30 cm in DEF, and 167 and 9.3 Mg/ha/30 cm in HEF, respectively.

### N Fluxes at DDF, DEF, and HEF

Tables 1 and 2 show the seasonal and vertical change of N fluxes in the soil at each site. In DDF, the high inorganic N production and uptake were exhibited in the surface soil horizon in the onset of wet season (Table 1 and 2). N production reached almost 70% of the annual production and 78% of the estimated annual uptake. In the late wet season, the produced inorganic N probably leached down to the deeper soil horizons.

In the DEF, inorganic N produced in the whole soil horizon in the early wet season (Table 1 and 2) was about 50% of the annual N production. In the early wet season, 67% of the estimated annual uptake was observed. In the HEF, produced inorganic N was taken up in all soil horizons throughout the wet season, whereas in the HEF, N fluxes fluctuated with the least N leaching (<5 kg/ha/year) of all the sites.

**TABLE 1**
**Seasonal Change of N Fluxes in Three Contrasting Dry Tropical Forests**

| Type of Forest | Type of N Flux | N Flux (kg N ha$^{-1}$) Sep.–Nov. 98 | Nov.–May 99 | May–Sep. 99 | Sep.–Nov. 99 |
|---|---|---|---|---|---|
| DDF | Production | — | 30.0 | 2.9 | 15.8 |
| | Leaching | 25.3 | 2.5 | 3.7 | 29.3 |
| | Pool change | 23.3 | 11.1 | 10.4 | 10.7 |
| | Estimated N uptake | — | 34.2 | 1.5 | 8.3 |
| DEF | Production | — | 57.1 | 22.9 | 37.3 |
| | Leaching | 11.2 | 7.2 | 1.9 | 41.8 |
| | Pool change | 18.5 | 15.2 | 12.8 | 33.8 |
| | Estimated N uptake | — | 52.7 | 23.6 | 3.5 |
| HEF | Production | — | 45.3 | 37.3 | 33.0 |
| | Leaching | 1.3 | 0.3 | 3.6 | 6.3 |
| | Pool change | 8.7 | 12.7 | 7.3 | 7.7 |
| | Estimated N uptake | — | 45.2 | 38.0 | 26.3 |

**TABLE 2**
**Vertical Change of N Fluxes in Three Contrasting Dry Tropical Forests**

| | | N Flux (kg N $ha^{-1}$ $horizon^{-1}$) | | |
|---|---|---|---|---|
| **Type of Forest** | **Type of N Flux** | **0–5 cm** | **5–10 cm** | **10–30 cm** |
| DDF | Production | 28.6 | 11.4 | 4.3 |
| | Leaching | 26.0 | 22.6 | 35.5 |
| | Pool change | –5.2 | –3.7 | –3.7 |
| | Estimated N uptake | 24.9 | 16.9 | 2.2 |
| DEF | Production | 30.9 | 15.3 | 67.0 |
| | Leaching | 26.9 | 44.6 | 50.9 |
| | Pool change | –0.3 | –1.2 | 16.9 |
| | Estimated N uptake | 10.7 | 8.3 | 59.8 |
| HEF | Production | 23.5 | 20.8 | 64.6 |
| | Leaching | 12.0 | 9.4 | 10.1 |
| | Pool change | 0.6 | –0.9 | –0.6 |
| | Estimated N uptake | 16.5 | 22.4 | 49.7 |

### DISCUSSION

#### C and N Accumulation in Dry Tropical Soil

In this study, soil C and N concentrations in the DDF and DEF were one order of magnitude lower than those of the temperate forests, but the pools were about one fifth of the temperate forests[15] (Fig. 1). The high fine soil content offset low C concentration. This study was done at only two sites, but the high fine soil content seems to be important for nutrient accumulation in highly weathered tropical soils. In the HEF, high C and N accumulation was observed and C and N concentrations were similar to those of the temperate forest[15]. Furthermore, the accumulation of thick forest floor (mor type forest floor) at the ridge, and the absence of such forest floor accumulation in DDF and DEF, may be one reason for this difference. The HEF is, therefore, considered the transition forest type from dry tropical to the temperate forests.

#### Seasonal and Vertical Difference in N Cycling

One interesting feature of the soil N dynamics is the seasonal difference in inorganic N flux among the sites (Table 1). With the highest biological activity, the DDF estimated N uptake was observed in the onset of wet season, and the estimated uptake significantly decreased in the mid-wet season ($p = 0.05$). In contrast, biological activity was highest in the early wet season in DEF and HEF, whereas the estimated uptake was relatively stable throughout the wet season ($p > 0.8$). N cycling in dry tropical forest requires long periods of monitoring under different forest types. This study was carried out in only three forests, and therefore further investigation is required to completely understand N cycling patterns in these forests. However, the trend in which N cycling becomes stable from DDF to HEF and the stable N uptake in HEF is noteworthy and consistent with other studies[5].

Furthermore, there were clear vertical differences in soil N dynamics among sites (Table 2). In the DDF, the N cycle was relatively high in the surface horizon (0 to 5 cm), whereas N fluxes in DEF and HEF were significantly higher in the deeper soil horizon (>10 cm). Inorganic N was mainly produced at 10- to 30-cm soil horizon in the DEF and HEF, and the inorganic N was significantly greater than in the upper soil horizon ($p < 0.03$). The deeper horizons were important for N cycling in DEF and HEF and correspond with high C content in deeper horizons (Fig. 1). N cycling in the whole soil horizons is likely to stabilize N cycling. Soil parent materials at the study sites are different, and since soil properties are strongly controlled by the properties of their parent materials, it is impossible for us to determine how differences in soil properties among sites impact their N cycling patterns.

Spatial patterns of N cycling may be related to forest type. Flux of N leaching from 30-cm soil depth was significantly smaller in HEF than in DDF and DEF ($p < 0.01$, Table 2). In terms of N leaching, it seems to be relatively conservative and closer N cycle in HEF compared to DDF and DEF. This study shows that N deposition may result in the large changes of N cycling in the dry tropical forests through low accumulation of C and N in the DDF and DEF.

### ACKNOWLEDGEMENTS

We would like to thank the students of Kasetsert University for their support in the fieldwork and Ms. Tsukura and Ms. Yamamoto for the laboratory work. We also thank Mr. Yamamoto of Organo Chemical Company for providing ion resin. Financial support for this project came from the International Geosphere-Biosphere Project (Ministry of Education).

| | | |
|---|---|---|
| Received: | July | 28, 2001 |
| Revised: | November | 9, 2001 |
| Accepted: | November | 9, 2001 |
| Published: | November | 20, 2001 |
